# TaWRKY31, a novel WRKY transcription factor in wheat, participates in regulation of plant drought stress tolerance

**DOI:** 10.1186/s12870-023-04709-7

**Published:** 2024-01-03

**Authors:** Miaomiao Ge, Yan Tang, Yijun Guan, Meicheng Lv, Chunjv Zhou, Huiling Ma, Jinyin Lv

**Affiliations:** College of Life Sciences, Northwest A&F University, Yangling, China

**Keywords:** Wheat (*Triticum aestivum* L.), Drought stress tolerance, *TaWRKY31*, Molecular mechanism, Transcriptional regulation

## Abstract

**Background:**

Wheat, a crucial food crop in China, is highly vulnerable to drought stress throughout its growth and development. WRKY transcription factors (TFs), being one of the largest families of TFs, play a vital role in responding to various abiotic stresses in plants.

**Results:**

Here, we cloned and characterized the TF TaWRKY31 isolated from wheat. This TF, belonging to the WRKY II family, contains a WRKYGQK amino acid sequence and a C_2_H_2_-type zinc finger structure. *TaWRKY31* exhibits tissue-specific expression and demonstrates responsiveness to abiotic stresses in wheat. TaWRKY31 protein is localized in the nucleus and can function as a TF with transcription activating activity at the N-terminus. Results showed that the wheat plants with silenced strains (BSMV:*TaWRKY31*-1as and BSMV:*TaWRKY31*-2as) exhibited poor growth status and low relative water content when subjected to drought treatment. Moreover, the levels of O_2_·^−^, H_2_O_2_, and malondialdehyde (MDA) in the BSMV:*TaWRKY31*-induced wheat plants increased, while the activities of antioxidant enzymes (superoxide dismutase, peroxidase, and catalase) decreased. Compared to control plants, BSMV:*TaWRKY31*-induced wheat plants exhibited lower expression levels of *TaSOD (Fe)*, *TaPOD*, *TaCAT*, *TaDREB1*, *TaP5CS*, *TaNCED1*, *TaSnRK2*, *TaPP2C*, and *TaPYL5*.Under stress or drought treatment conditions, the overexpression of *TaWRKY31* in *Arabidopsis* resulted in decreased levels of H_2_O_2_ and MDA, as well as reduced stomatal opening and water loss. Furthermore, an increase in resistance oxidase activity, germination rate, and root length in the *TaWRKY31* transgenic *Arabidopsis* was observed. Lastly, overexpression of *TaWRKY31* in *Arabidopsis* resulted in higher the expression levels of *AtNCED3*, *AtABA2*, *AtSnRK2.2*, *AtABI1*, *AtABF3*, *AtP5CS1*, *AtSOD (Cu/Zn)*, *AtPOD*, *AtCAT*, *AtRD29A*, *AtRD29B*, and *AtDREB2A* than in control plants.

**Conclusions:**

Our findings indicate that *TaWRKY31* enhances drought resistance in plants by promoting the scavenging of reactive oxygen species, reducing stomatal opening, and increasing the expression levels of stress-related genes.

**Supplementary Information:**

The online version contains supplementary material available at 10.1186/s12870-023-04709-7.

## Background

Drought is the primary detrimental abiotic stress in plant agriculture, severely affecting the crop growth, development, and yield [1]. As one of the three primary cereals, wheat (*Triticum aestivum* L.) is the most widely cultivated cereal in the world, contributing more than 60% to people’s daily calorific and protein requirements [2]. However, its yield is severely affected by abiotic and biotic stresses. Over time, plants have evolved a series of complex strategies to survive under climatic stress (e.g., drought stress) through a wide range of morphological and physiological metabolic and molecular mechanisms [3]. Phytohormones play a significant role in abiotic stress tolerance; meanwhile, transcription factors (TFs), such as NAC, bHLHs, DREBs, MYB proteins, C_2_H_2_-type zinc fingers, and WRKY, are involved in the regulation of abiotic stress in plants. Previous studies have shown that the heterologous overexpression of *SlNAC8* in *Arabidopsis* enhances plant tolerance to drought and salt stress by altering their physiological and biochemical characteristics. This is achieved by regulating the expression of stress-responsive genes including *RD20*, *GSTF6*, *COR47*, *RD29A*, *RD29B*, and *NYC1* [4]. Moreover, the expression levels of abiotic stress/abscisic acid (ABA)-related genes in transgenic *Arabidopsis* have been reported to increase under drought and salt stress conditions. Wu et al. reported *ZmMYB3R* to be a positive TF that enhances plant tolerance to drought and salt stress through an ABA-dependent pathway [5]. *TaMpc1-D4*, which encodes an R2R3 MYB TF located on chromosome D of wheat, negatively regulates drought tolerance in transgenic *Arabidopsis* and wheat by controlling the expression levels of reactive oxygen species (ROS), antioxidants, and ABA-related genes [6]. Moreover, in transgenic *Arabidopsis*, *PwNAC11* enhances drought resistance by physically interacting with the ABA-responsive element (ABRE)-binding factor 3 (ABF3) and dehydration-responsive element (DRE)-binding protein 2 A (DREB2A) for activating the expression of the downstream gene *ERD1* [7]. *AgMYB5*, on the other hand, triggers the expression of *AtCRTISO* and *AtLCYB*, which promotes β-carotene biosynthesis.

WRKY proteins are plant-specific TFs that play a crucial role in plant growth, development, and stress resistance. To date, a total of 124 WRKY genes, including 294 homologous copies, have been identified in wheat [8]. However, relatively few members of *TaWRKY* involved in drought have been examined. WRKY TFs are characterized based on their N-terminal region, which contains a conserved amino acid sequence known as the WRKY domain. This domain enables the TFs to bind to the W-box motif found in the promoters of target genes, thereby regulating their expression [9, 10]. The WRKY domain consists of 60 amino acid residues. In addition to the N-terminal WRKYGQK, its C-terminus also demonstrates an atypical zinc finger structure [11]. The number of WRKY domains in TFs and the characteristics of zinc finger structure divide WRKY TFs into three groups [10, 12].

Herein, we studied the TaWRKY31 TFs, which belong to the IIc group, and we also found that *TaWRKY31* was rapidly induced and its expression was significantly increased after water deficit stress in wheat [13]. WRKY TFs belonging to group II only contain a single WRKY domain, and their zinc finger structure is Cx_4 − 5_Cx_22 − 23_HxH. Additionally, most of these TFs studied herein belong to group II. According to the phylogenetic relationship of the amino acid sequence in the primary structure, group II can be further categorized into IIa, IIb, IIc, IId, and IIe subgroups [11, 14–16]. The WRKY TFs involved in plant responses to abiotic stresses have been extensively studied in several model plants. For instance, *NCED3* and *ABA3* encode key enzymes in the ABA synthesis pathway [17, 18]. AtWRKY57 TF directly interacts with the W-box of the *NCED3* promoter sequence, thereby activating *NCED3* expression and indirectly upregulating *ABA3* expression. Furthermore, *AtWRKY57* confers drought tolerance in transgenic *Arabidopsis* by increasing ABA levels [19]. Overexpressing *TaWRKY46* in *Arabidopsis* significantly increases the expression of stress-related genes such as *P5CS1*, *RD29B*, *DREB2A*, *ABF3*, *CBF2*, and *CBF3*, which confers drought tolerance to plants [20]. Moreover, *MaWRKY80* directly binds to the W-box in the promoter regions of *AtNCEDs* in *Arabidopsis* and *MaNCEDs* in bananas. It regulates the expression of 9-cis-epoxycarotenoid dioxygenases (NCEDs) and ABA biosynthesis, while also controlling ROS accumulation to enhance plant drought resistance [21].

In previous studies, the inhibition of water loss rate (WLR), electrolyte leakage, and ROS and malondialdehyde (MDA) accumulation has confirmed the enhanced drought tolerance of the *EjWRKY17* transgenic lines. Meanwhile, Wang et al. reported that *EjWRKY17* improves ABA-induced stomatal closure and activates the expression of stress-related genes [22]. TFs control plant’s key downstream responses by regulating the transcription of target genes, therefore emerging as major targets for improving plant stress resistance [23]. Previous studies have shown that numerous WRKY TFs are involved in ABA-mediated stress signal transduction [24]. The WRKY protein can function as both an activator and a repressor of ABA-inducible promoters in abiotic stress [25]. Although the role of WRKY TFs in regulating drought resistance has been sufficiently elaborated in *Arabidopsis* and rice, reports on wheat as a model plant are lacking, and the underlying molecular mechanisms remain unclear. Therefore, herein, we conducted sequence analysis of *TaWRKY31* and performed functional characterization of *Arabidopsis* overexpression and wheat gene silencing. Furthermore, the role of *TaWRKY31* in conferring drought resistance was determined, and the underlying mechanism was preliminarily studied and explored.

## Results

### Bioinformatics analysis of *TaWRKY31*

**Fig. 1:**
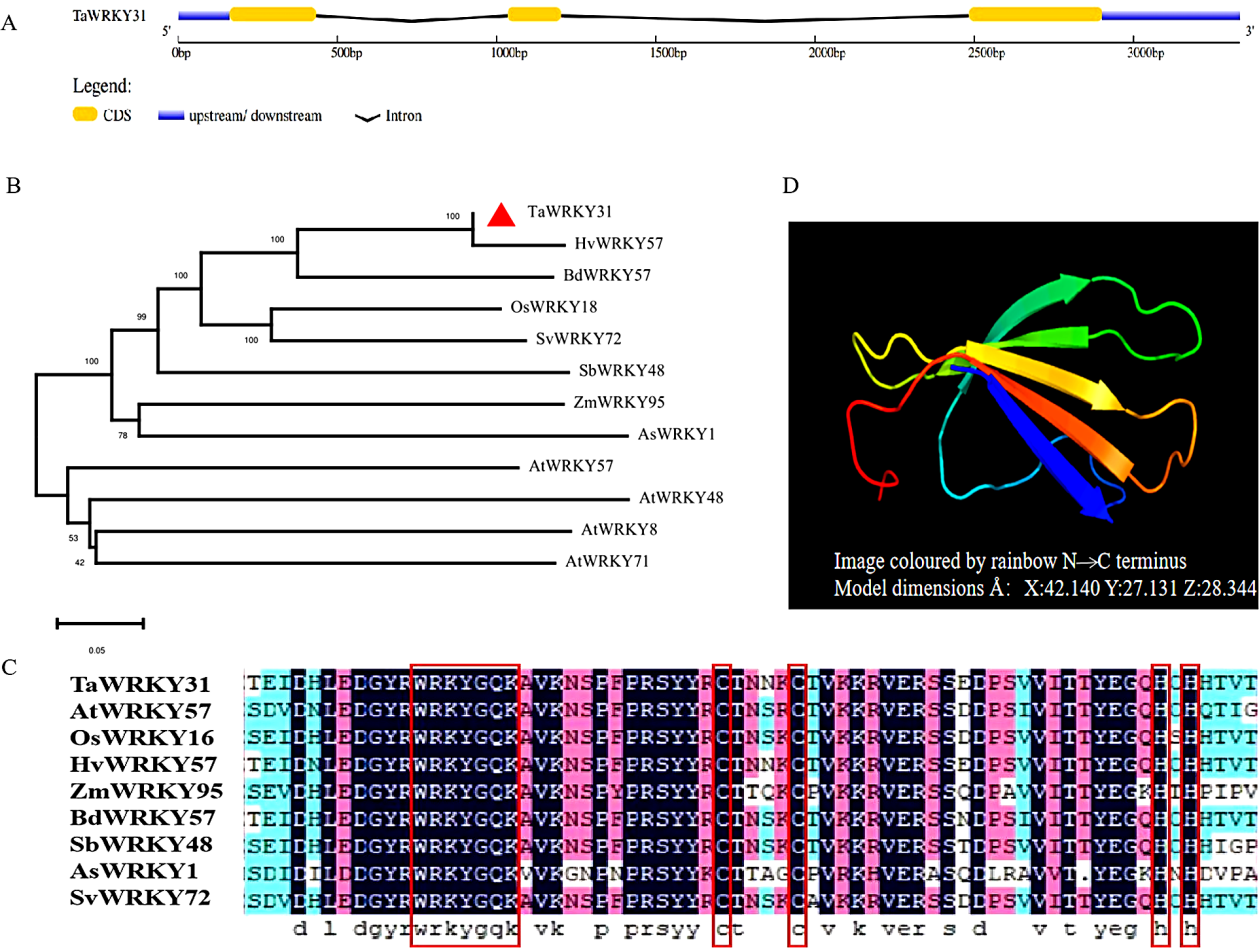
Analysis of TaWRKY31 in bioinformatics. **(A)** Schematic diagram of *TaWRKY31* gene structure. **(B)** Phylogenetic relationships of *TaWRKY31* with homologous sequences of other species. **(C)** Sequence comparison of TaWRKY31 and its homologous genes. Dark blue, pink, and light blue represent 100%, 75%, and 50% similarity, respectively. **(D)** Tertiary structure prediction model of TaWRKY31 protein

According to the Ensembl Plants database (plants.ensembl.org), specific primers were designed for amplifying the *TaWRKY31* open reading frame (TraesCS2B02G280300), which was cloned from a wheat cDNA library. *TaWRKY31* gene is 3333 bp in length and consists of three exons and two introns (Fig. 1A). Results showed that *TaWRKY31* encodes a protein of 285 amino acid residues, with the molecular weight (Mw) of the deduced protein being 30.339 kDa and the isoelectric point (pI) being 6.60. A phylogenetic tree was constructed to analyze the evolutionary relationship of TaWRKY31 protein with WRKY proteins from *Poaceae Barnhart* and *Arabidopsis thaliana*. The results showed that *HvWRKY57* (XM_045110161.1) in *Hordeum vulgare* exhibited a high sequence similarity and homology with *TaWRKY31*. The closest homolog of *TaWRKY31* in *Arabidopsis* was *AtWRKY57* (Fig. 1B). Further analysis revealed that the TaWRKY31 protein contains a typical WRKY domain, including the conserved amino acid sequence “WRKYGQK” and a C_2_H_2_ zinc finger motif (Fig. 1C), which is a characteristic feature of group II members of the WRKY protein family [10]. The TaWRKY31 protein was modeled using 75 amino acid residues, accounting for approximately 26% of the total protein sequence. The modeling was done with 100% confidence, with the N-terminal region represented in blue and the C-terminus in orange. Additionally, a tertiary structure model of the TaWRKY31 TF was generated, showing five β-sheets in the protein structure (Fig. 1D).

### Expression pattern of *TaWRKY31* under various abiotic stresses

**Fig. 2:**
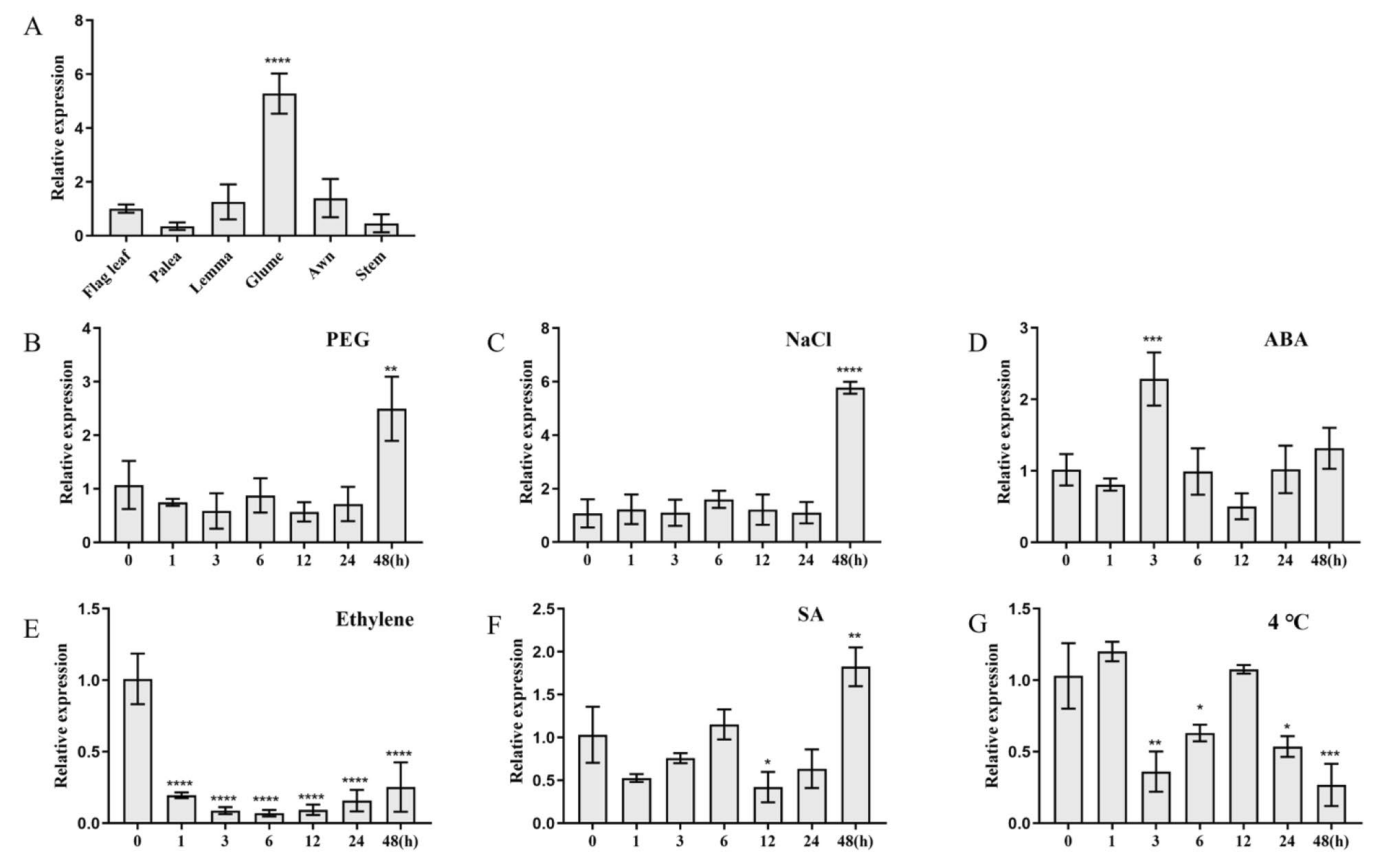
Expression pattern analysis of *TaWRKY31*. **(A)** Expression pattern analysis of *TaWRKY31* in different organs of wheat. Relative expression of *TaWRKY31* under **(B)** 20% polyethylene glycol (PEG), **(C)** 100 mM NaCl, **(D)** 100 µM abscisic acid (ABA), **(E)** 100 µL·L^− 1^ ethylene, **(F)** 500 µmol·L^− 1^ salicylic acid (SA), and **(G)** 4 °C treatments. Asterisks indicate significant differences (* p < 0.05, ** p < 0.01, *** p < 0.001, **** p < 0.0001). No asterisk indicates that the difference is not significant

The transcription level of *TaWRKY31* was analyzed in different wheat tissues, including the flag leaf, palea, lemma, glume, awn, and stem. *TaWRKY31* was found to be widely expressed in various tissues, with the highest transcript levels observed in glume and lower levels in palea (Fig. 2A). To understand the potential role of *TaWRKY31* in abiotic stress, its expression pattern was examined under different abiotic stress conditions using the qRT-PCR method. Under PEG-6000 and NaCl stresses, *TaWRKY31* expression did not change significantly at 0, 1, 3, 6, 12 and 24 h; however, it was significantly upregulated with the highest transcript levels at 48 h, with fold increases of 2.49 and 5.77 compared to those under control conditions, respectively (Fig. 2B, C). Under exogenous ABA, which is a phytohormone involved in salt and drought stress responses, *TaWRKY31* expression was significantly induced at 3 h, reaching a maximum level of approximately 2.28-fold (Fig. 2D). The expression of *TaWRKY31* was found to be inhibited under ethylene treatment (Fig. 2E). It showed slight fluctuations under SA treatment (Fig. 2F) and did not change significantly under low-temperature treatment (Fig. 2G). Based on these expression patterns, it was speculated that *TaWRKY31* might be closely related to osmotic stress.

### Subcellular localization and transcriptional activation activity of *TaWRKY31*

**Fig. 3:**
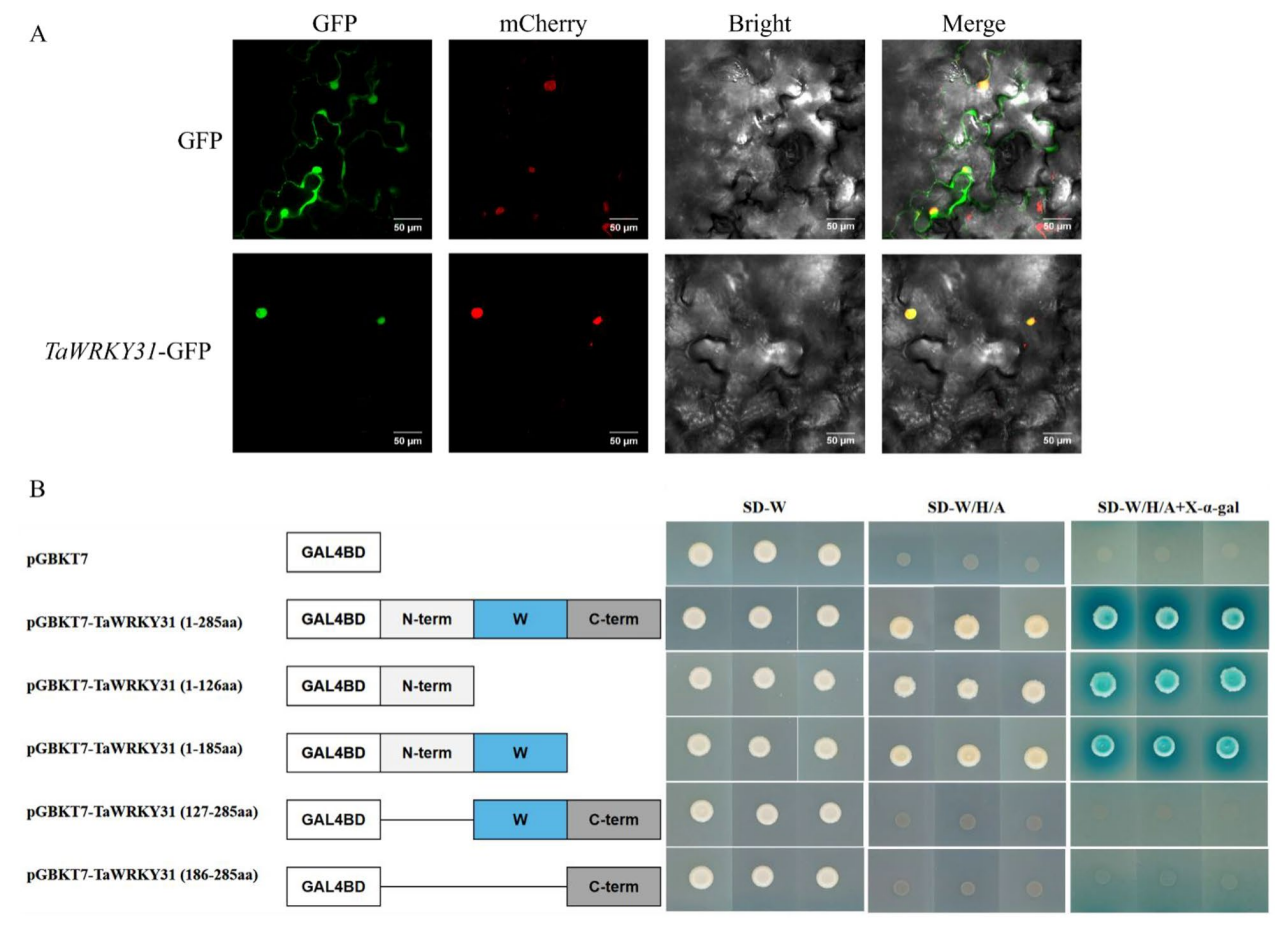
Subcellular location and transcriptional activation assay of *TaWRKY31*. **(A)** Subcellular localization of *TaWRKY31* in the tobacco epidermis. Fluorescence detection of tobacco leaf epidermal cells, with 35 S::GFP transformed tobacco epidermis as control; mCherry is the nuclear localization marker. Scale bar = 50 μm. **(B)** Schematic representation of different truncated fragments of TaWRKY31 ligated to the pGBKT7 vector. Yeast cells were cultured on selective medium without tryptophan (SD-W), SD-W medium lacking tryptophan, histidine, and adenine (SD-W/H/A), and SD-W/H/A + X-α-D-galactosidase (X-α-gal)

Cell-Ploc 2.0 (http://www.csbio.sjtu.edu.cn/bioinf/Cell-PLoc-2/) was utilized to predict the subcellular localization of *TaWRKY31*, and the results indicated that *TaWRKY31* is localized in the cell nucleus. To further validate the prediction, the coding sequence of *TaWRKY31*, excluding its stop codon, was fused to the 5’-terminus of GFP in p35s-1301-GFP vector, under the control of the cauliflower mosaic virus (CaMV) 35 S promoter. The resulting fusion protein, 35 S::TaWRKY31-GFP, was expressed in the lower epidermis of tobacco plants to visualize its subcellular location. Scanning confocal microscopy revealed that the control group expressing 35 S::GFP exhibited fluorescence in the entire cells, whereas green fluorescence was specifically observed in the nucleus of 35 S::TaWRKY31-GFP transformed cells, aligning with the nuclear localization marker position (Fig. 3A). These findings provide further evidence supporting the nuclear localization of *TaWRKY31*.

To identify the region responsible for self-activation activity in the TaWRKY31 protein, we divided the full-length TaWRKY31 ORF into four different fragments: TaWRKY31-N (1-126aa), TaWRKY31-NW (1-185aa), TaWRKY31-WC (127-285aa), and TaWRKY31-C (186-285aa). These fragments, along with the complete TaWRKY31 ORF (1-285aa), were cloned into the pGBKT7 vector and transformed into the yeast strain AH109, with the pGBKT7 empty vector serving as a negative control. Yeast cells harboring any of these six vectors cultured well on SD-W medium. Moreover, yeast cells containing TaWRKY31, TaWRKY31-N, and TaWRKY31-NW vectors exhibited good growth on the selection medium SD-W/H/A and displayed a blue color indicative of α-galactosidase activity. In contrast, yeast cells containing TaWRKY31-WC, TaWRKY31-C, and the pGBKT7-empty vector showed abnormal growth (Fig. 3B). These results suggest that TaWRKY31 protein possesses self-transcriptional activation activity in yeast cells, and deletion of the N-terminal region leads to the loss of this function.

### Effects of *TaWRKY31* silencing in wheat under drought treatment

**Fig. 4:**
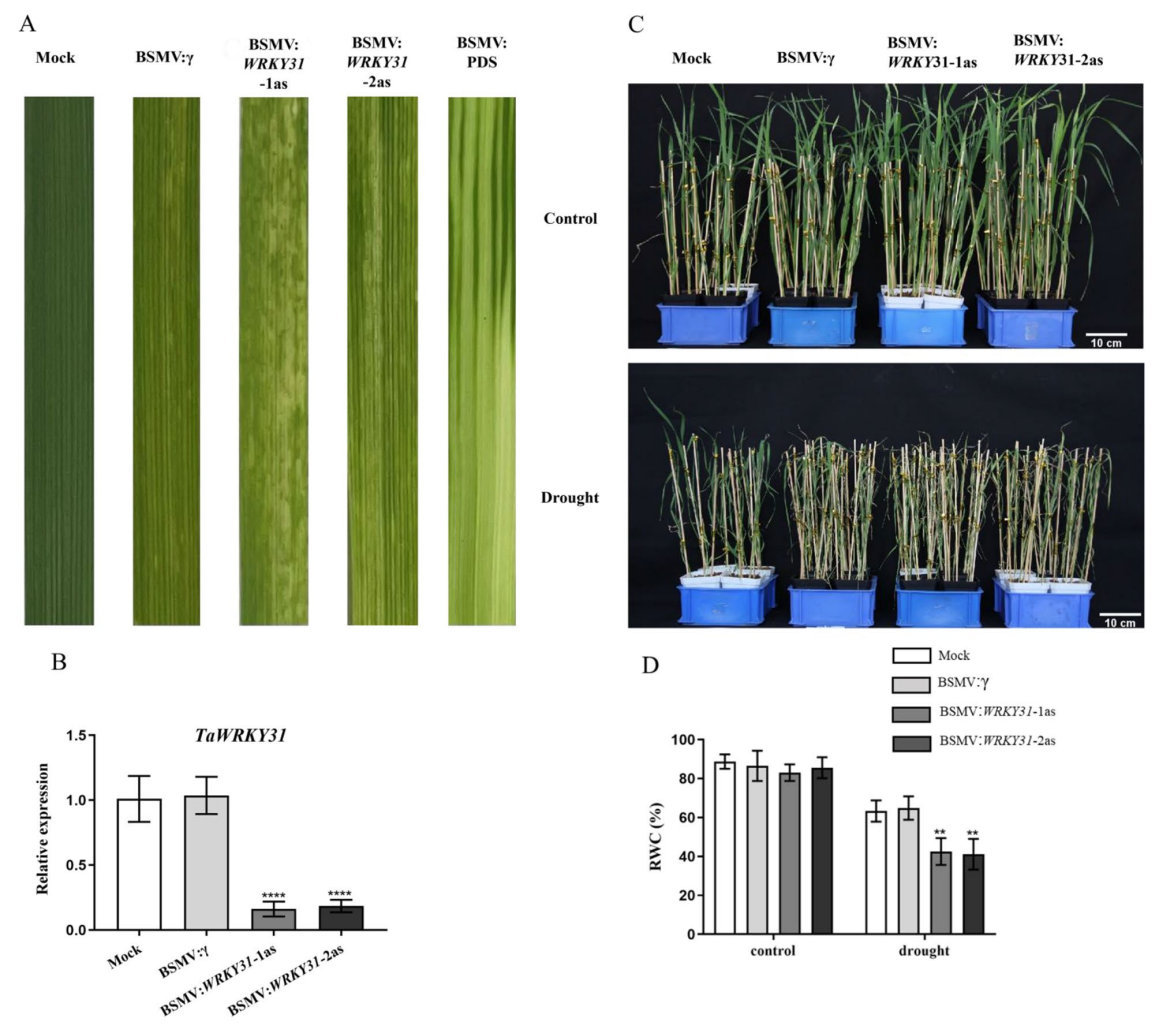
Silencing efficiency and phenotypic analysis of *TaWRKY31*-silenced strains under drought stress in wheat. **(A)** Phenotype of *TaWRKY31*-silenced wheat leaves. **(B)** The silencing efficiency of *TaWRKY31*-silenced lines before drought treatment in wheat. **(C)** Phenotypic analysis of *TaWRKY31*-silenced lines after 10 d of drought treatment in wheat; the upper image depicts the plants subjected to control treatment, while the lower image depicts the plants subjected to drought treatment. **(D)** Relative water content (RWC) of *TaWRKY31*-silenced lines after 10 d of drought treatment in wheat. Asterisks indicate significant differences between lines under the same treatment (** p < 0.01, **** p < 0.0001)

To investigate the phenotypic implications of *TaWRKY31* silencing in wheat under abiotic stress, we employed the BSM VIGS system to suppress the expression of *TaWRKY31*. The third leaf of wheat seedlings was observed after inoculating the virus on the second leaf for 10 d, and the third leaf was allowed to grow for 10 d in a high temperature and humidity environment. Wheat plants inoculated with BSMV:TaPDS (wheat phytoene desaturase gene) served as a positive control, showing significant photobleaching of the leaves. Plants inoculated with the FES buffer (Mock) did not exhibit significant changes and had green leaves. In contrast, leaves of the empty vector (BSMV-γ), BSMV:*WRKY31*-1as, and BSMV:*WRKY31*-2as lines displayed mild chlorotic mosaic symptoms without evident defects (Fig. 4A). Furthermore, qRT-PCR analysis confirmed approximately 84% silencing efficiency of the *TaWRKY31* gene (Fig. 4B). These results indicate successful operation of the VIGS system, resulting in two wheat lines BSMV:*WRKY31*-1as and BSMV:*WRKY31*-2as with silenced *TaWRKY31* gene expression.

Following 20 d of growth, the wheat seedlings were subjected to a 10-d drought treatment. Under drought stress, the BSMV:*WRKY31*-1as and BSMV:*WRKY31*-2as plants exhibited mild wilting and yellowing, with leaf growth worse compared to that of the Mock seedlings (Fig. 4C). The RWC of the two *WRKY31*-silenced lines was significantly decreased under drought stress (Fig. 4D). These observations suggest that gene silencing of *TaWRKY31* reduces the drought tolerance of plants.

### BSMV-mediated *TaWRKY31* silencing decreased drought tolerance

**Fig. 5:**
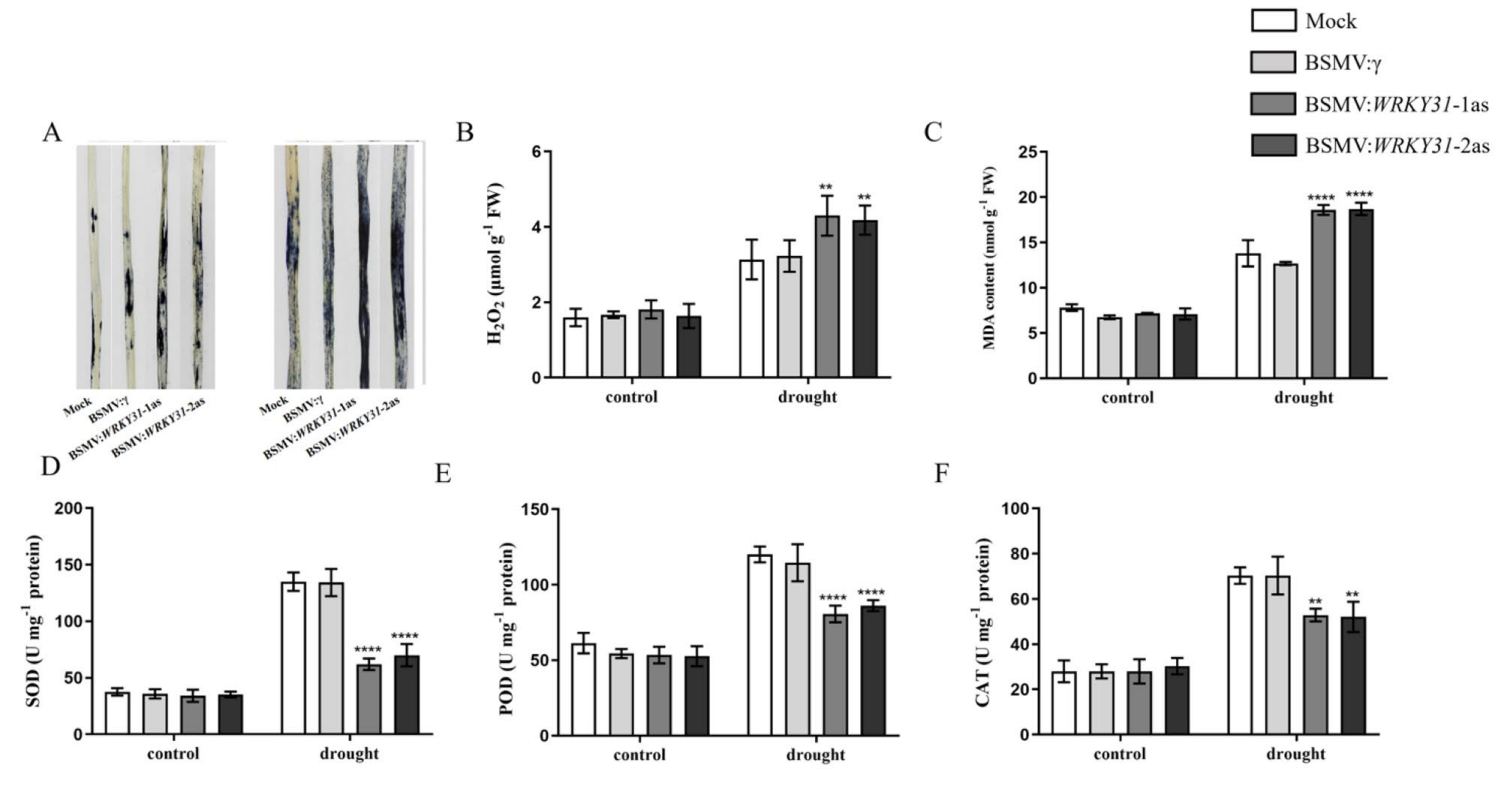
Effect of drought stress on O_2_·^−^, malondialdehyde (MDA), H_2_O_2_, as well as peroxidase (POD), superoxide dismutase (SOD), and catalase (CAT) activities in *TaWRKY31*-silenced lines of wheat. **(A)** Histochemical staining of O_2_·. **(B)** H_2_O_2_ content. **(C)** MDA content. **(D)** SOD activity. **(E)** POD activity. **(F)** CAT activity. Asterisks indicate significant differences between lines under the same treatment (** p < 0.01, *** p < 0.001, **** p < 0.0001)

When exposed to abiotic stress, plant cells produce excessive ROS, such as hydrogen peroxide (H_2_O_2_) and superoxide anion radical (O_2_·^−^). To assess the level of ROS in plant cells under drought stress, histochemical staining using NBT was performed. Under normal conditions (CK), only a few leaves from the four strains exhibited blue staining. However, after drought treatment, almost all leaves of BSMV:*WRKY31*-1as and BSMV:*WRKY31*-2as displayed blue staining, while the leaves of Mock and BSMV:γ strains showed relatively minimal staining (Fig. 5A). The H_2_O_2_ content in the leaves was further quantified, and no significant difference was observed among all leaves under normal conditions. However, after drought stress treatment, the H_2_O_2_ content in BSMV:*WRKY31*-1as and BSMV:*WRKY31*-2as plants was higher than that in Mock and BSMV:γ strains (Fig. 5B), indicating that the gene-silenced wheat plants accumulated more ROS in comparison to Mock and BSMV:γ plants. Additionally, the MDA content, which reflects the severity of lipid oxidation, was significantly increased in the leaves of BSMV:*WRKY31*-1as and BSMV:*WRKY31*-2as strains (Fig. 5C). Furthermore, assays of antioxidant enzyme activities revealed that the activities of SOD, POD, and CAT were significantly lower in the gene-silenced strains during drought treatment compared with those under normal conditions (Fig. 5D–F). These findings indicate that wheat seedlings are subjected to greater drought stress when the *TaWRKY31* gene is silenced, thereby reducing plant stress tolerance.

### Plant drought tolerance is reduced by altering the expression of antioxidant enzymes and stress response genes in *TaWRKY31*-silenced wheat lines

**Fig. 6:**
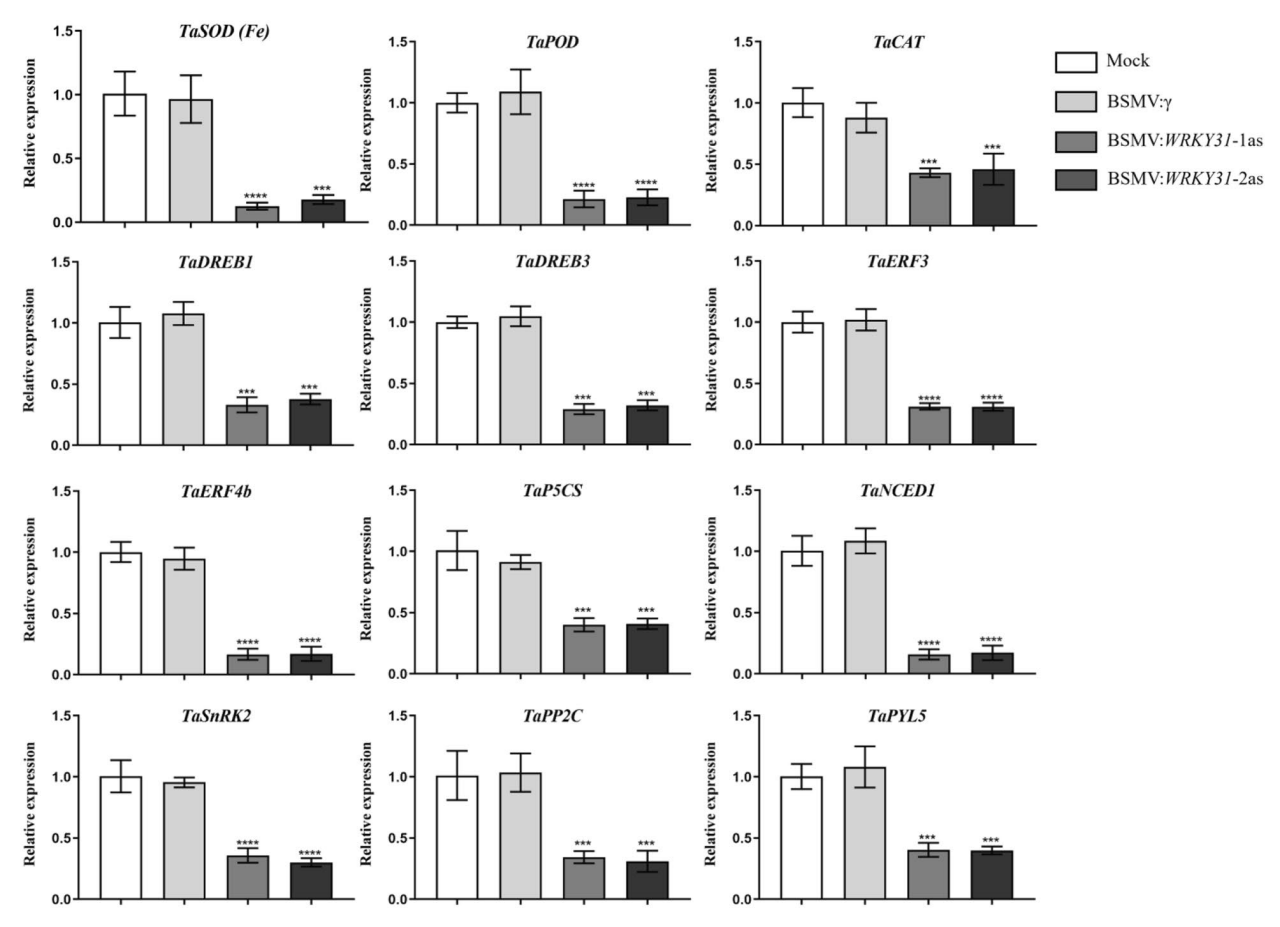
Transcriptional profiles of stress-related genes in *TaWRKY31*-silenced strains of wheat after drought treatment. Asterisks indicate significant differences between lines under the same treatment (*** p < 0.001, **** p < 0.0001)

We found that plant antioxidant enzyme activities were enhanced under drought stress. Further, qRT-PCR analysis to determine the expression of antioxidant enzyme-related genes, including *TaSOD (Fe)*, *TaPOD*, and *TaCAT* revealed that these genes were significantly downregulated in *TaWRKY31*-silenced plants compared to that in Mock and BSMV:γ plants. Additionally, we examined the transcriptional profiles of *TaP5CS*, *TaDREB1*, *TaDREB3*, ethylene-response factor 3 *(TaERF3)*, *TaERF4b*, *TaNCED1*, *TaPYL5*, *TaSnRK2*, and *TaABF* genes, all of which demonstrated various degrees of downregulation (Fig. 6). These results suggest that the *TaWRKY31*-silenced strains reduced the drought resistance of wheat seedlings by downregulating the expression profile of stress-related genes.

### Overexpression of *TaWRKY31* increases drought tolerance in transgenic *Arabidopsis*

**Fig. 7:**
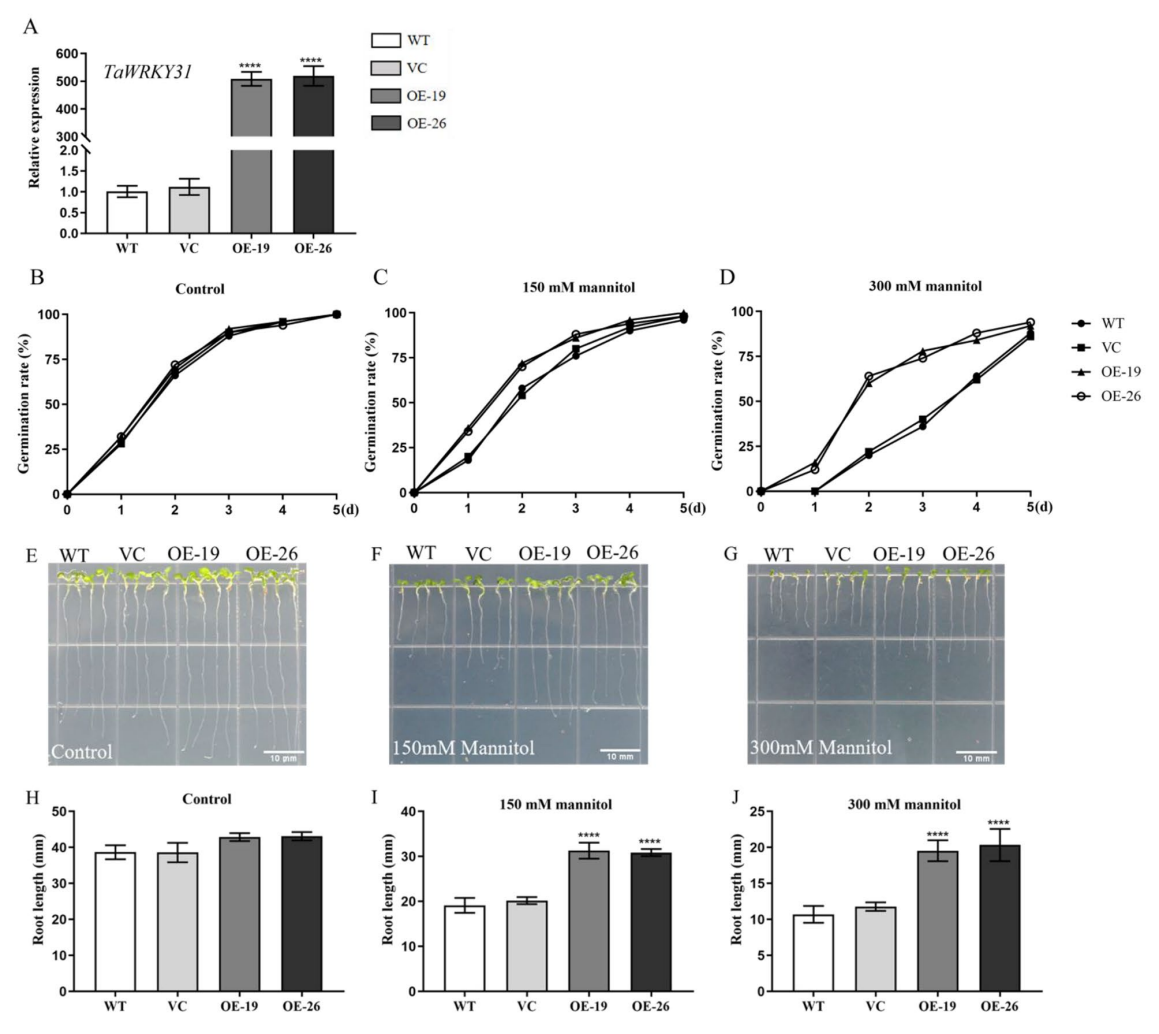
Effect of mannitol treatment on *TaWRKY31* overexpression in *Arabidopsis*. **(A)** Gene verification of *TaWRKY31* in overexpressed (OE) lines by RT-PCR. **(B, C,** and **D)** Germination of *TaWRKY31* OE lines on 1/2 MS plates containing mannitol (0, 150, and 300 mM). **(E-J)** The statistics of root length after mannitol treatments (0, 150, and 300 mM) for 5 d. Asterisks indicate the significant difference among WT, VC, and OE lines under the same treatment (**** p < 0.0001). Scale bar represents 10 mm

In order to investigate the potential role of *TaWRKY31* in drought stress tolerance, we conducted heterologous overexpression of the *TaWRKY31* gene in *Arabidopsis*. Two homozygous T_3_ transgenic *Arabidopsis* lines were generated and confirmed by kanamycin resistance screening and qRT-PCR analysis, which showed significantly higher expression levels of *TaWRKY31* in the two transgenic lines than in the WT and VC plants (Fig. 7A).

To assess the osmotic stress tolerance of the transgenic *Arabidopsis* lines, we performed seed germination assays on 1/2 MS solid medium supplemented with different concentrations of mannitol (0, 150, and 300 mM) and monitored the seed germination rate for 5 d. We observed no significant difference in seed germination among the four strains of *Arabidopsis* (WT, VC, OE-19, and OE-26) on the medium without mannitol and noted that all seeds from the four strains germinated within 5 d (Fig. 7B). However, under the presence of 150 mM mannitol, the germination rate of the transgenic seeds (OE-19 and OE-26) was significantly higher than that of WT and VC during the first three days (Fig. 7C). Moreover, in the presence of 300 mM mannitol, the germination rates of WT and VC seeds were 88% and 86%, respectively, while those of OE-19 and OE-26 seeds were both above 90% (Fig. 7D).

Furthermore, to determine the association of *TaWRKY31* with drought tolerance, we examined the root length of *Arabidopsis* seeds from the four strains (WT, VC, OE-19, and OE-26) on 1/2 MS solid medium supplemented with different concentrations of mannitol (0, 150, and 300 mM). Under normal conditions, no significant difference in root length was observed among WT, VC, and transgenic plants. However, compared to WT and VC, the transgenic strains exhibited significantly longer roots under low concentrations of mannitol (150 mM). This difference became more pronounced when a higher concentration of mannitol (300 mM) was applied (Fig. 7E–J).

### *TaWRKY31* overexpression inhibits stomatal opening and water loss

**Fig. 8:**
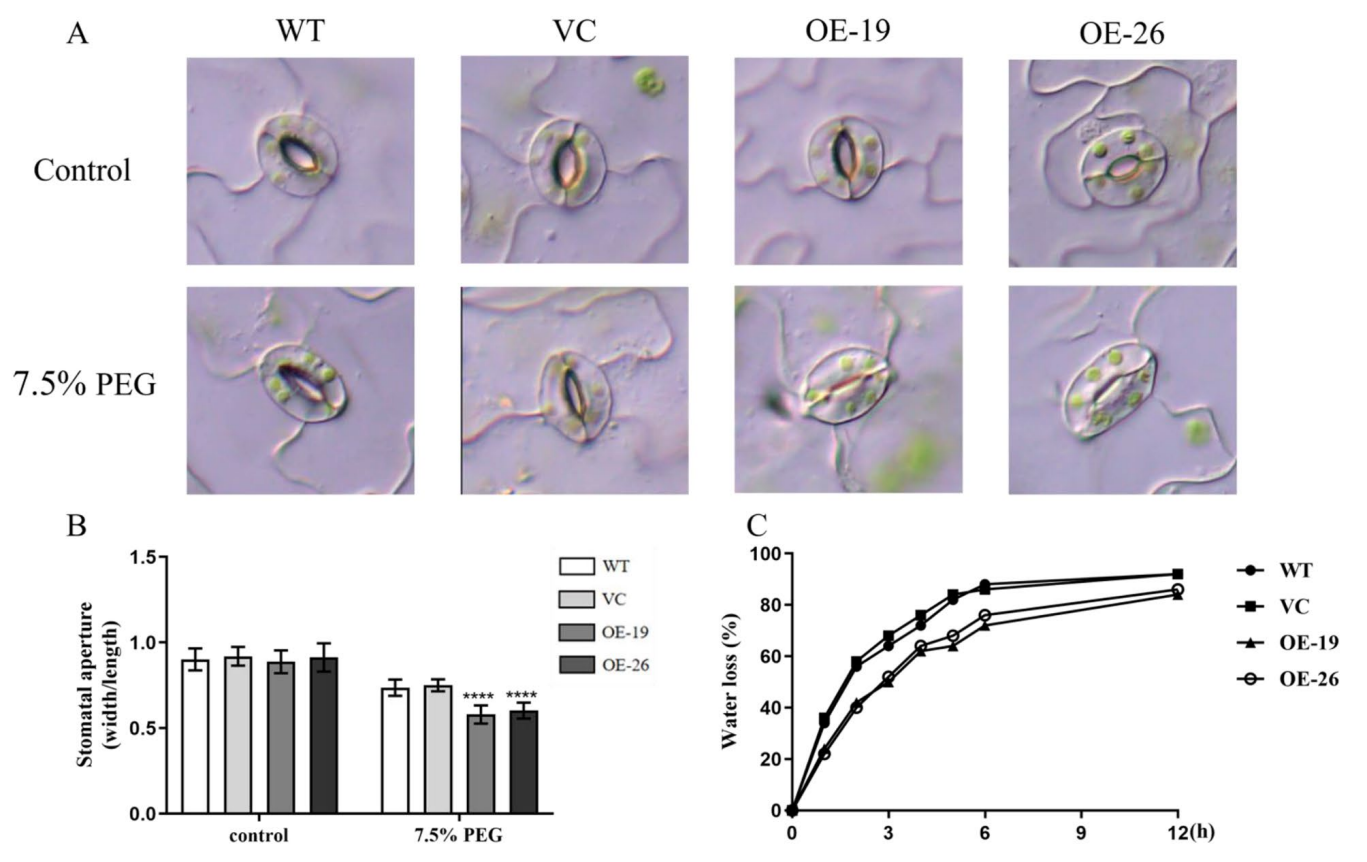
Phenotypes of *TaWRKY31* overexpressing *Arabidopsis* after drought treatment. **(A** and **B)** Stomatal aperture of transgenic *Arabidopsis* under 7.5% PEG treatment. **(C)** Water loss rate of transgenic *Arabidopsis* seedlings. Asterisks indicate significant differences (**** p < 0.0001)

Stomatal movement plays a crucial role in the survival of plants under drought conditions by regulating transpiration. Herein, we examined stomatal closure of leaves when exposed to 7.5% PEG. Under normal conditions, the stomata of plants from all four strains (WT, VC, OE-19, and OE-26) were predominantly open, and no significant difference was observed in stomatal aperture ratio between the transgenic plants and the WT and VC plants (Fig. 8A). However, after treatment with 7.5% PEG, the stomatal aperture (width/length ratio) in the transgenic strains was significantly smaller than that in the WT and VC plants. The stomatal aperture decreased to 0.58 and 0.60 in the two transgenic strains, respectively, being significantly lower than that in the WT and VC strains (Fig. 8B). Furthermore, we measured the WLR of isolated rosette leaves at the indicated time intervals. As shown in Fig. 8C, the WLRs of both OE-19 and OE-26 strains were significantly lower than those of WT and VC plants, except at 0 h. However, no significant difference was observed in the WLRs of leaves of OE-19 and OE-26 strains, indicating that overexpression of *TaWRKY31* leads to a slower rate of water loss from the leaves.

### Overexpression of *TaWRKY31* alters the physiological and biochemical properties of transgenic plants

**Fig. 9:**
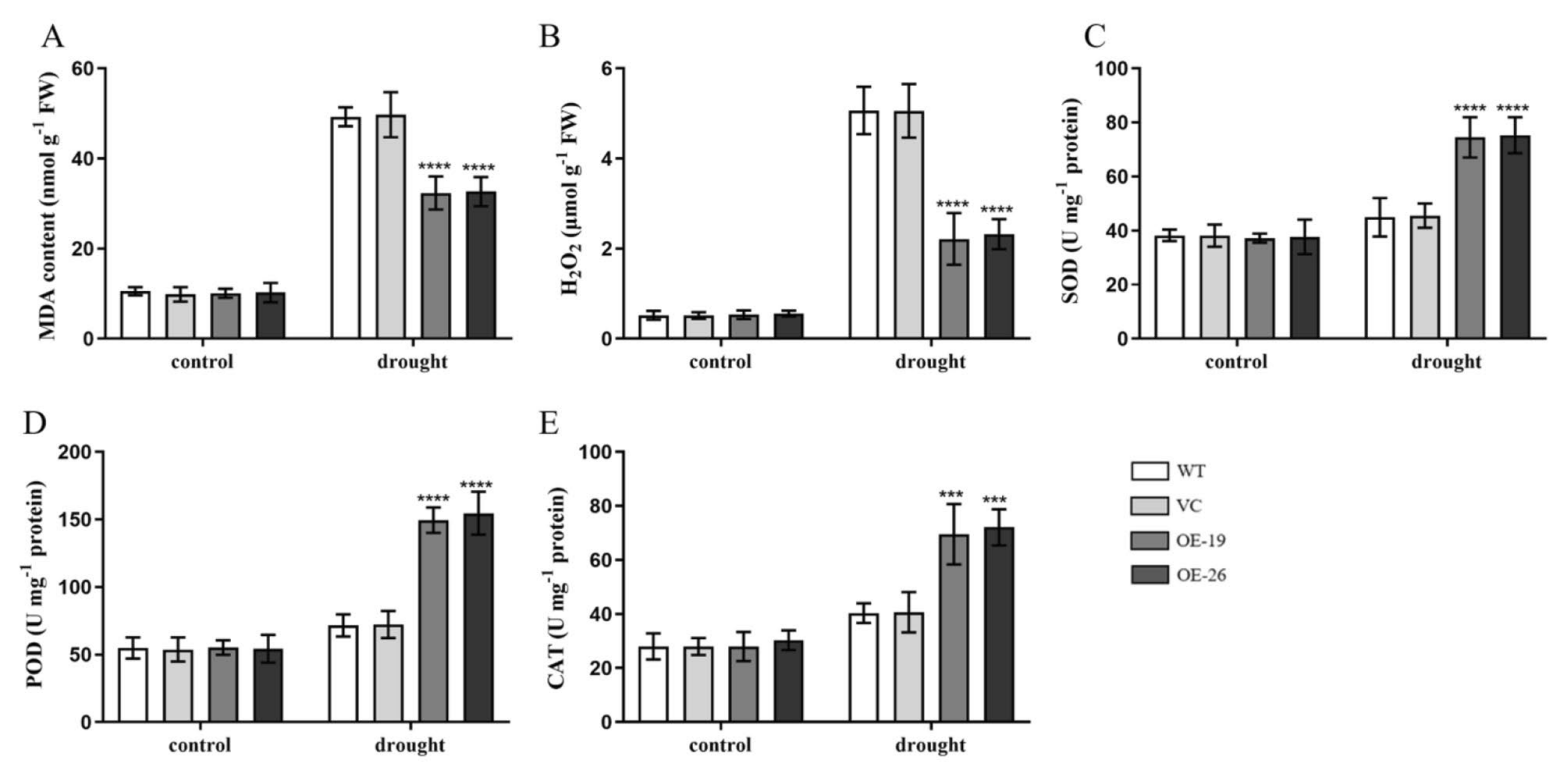
Effects of drought treatment on the contents of H_2_O_2_ and MDA and the activities of SOD, POD, and CAT in the *TaWRKY31*-overexpressing transgenic *Arabidopsis*. **(A)** MDA content. **(B)** H_2_O_2_ content. **(C)** SOD activity. **(D)** POD activity. **(E)** CAT activity. Asterisks indicate significant differences among different lines under the same treatment (*** p < 0.001, **** p < 0.0001)

To further validate the role of *TaWRKY31* in enhancing drought stress resistance in transgenic *Arabidopsis*, physiological parameters of WT, VC, and transgenic plants were analyzed under both 10 d of drought stress and normal water conditions. One of the indicators of oxidative damage in plant cells under stress conditions is the content of MDA, a product of membrane lipid peroxidation. Under normal conditions, no significant difference was observed in MDA content among WT, VC, and transgenic strains. However, under drought stress, the MDA content increased in all strains. Remarkably, the MDA content in the transgenic lines, OE-19 and OE-26, was approximately 0.65 times that of WT and VC (Fig. 9A).

To investigate whether *TaWRKY31* contributes to the degradation of excessive H_2_O_2_ in leaves, we quantified the H_2_O_2_ content in the leaves. Under normal conditions, all four strains (WT, VC, OE-19, and OE-26) exhibited extremely low levels of H_2_O_2_. However, after drought treatment, the H_2_O_2_ content increased in all strains, with transgenic plants showing approximately 43.7% greater content than that observed in WT and VC plants (Fig. 9B). Furthermore, we measured SOD, POD, and CAT activities. Under drought conditions, the activities of SOD, POD, and CAT were significantly higher in OE-19 and OE-26, being 1.64, 2.06, and 1.77 times higher than those in WT and VC, respectively. However, we observed no significant difference in these activities between OE-19 and OE-26 transgenic lines (Fig. 9C–E). These findings suggest that transgenic plants may enhance drought tolerance by regulating the ROS system.

### Overexpression of *TaWRKY31* improves the transcription levels of stress response genes

**Fig. 10:**
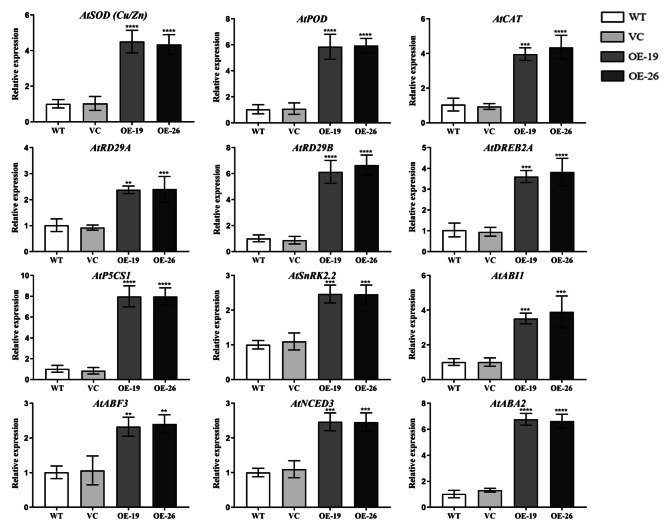
The transcriptional profiles of stress-related genes in *TaWRKY31* transgenic *Arabidopsis* lines under drought stress. Asterisks indicate the significant difference among WT, VC, and OE lines after drought treatment (* p < 0.05; ** p < 0.01; *** p < 0.001)

To investigate the potential molecular mechanisms underlying the role of *TaWRKY31* in response to drought stress, we examined the relative expression levels of genes associated with stress response in WT, VC, and transgenic *Arabidopsis*. We further analyzed the expression of several genes involved in different pathways. These included genes that are involved in ABA biosynthesis (*AtNCED3* and *AtABA2*), ABA signaling (*AtSnRK2.2*, *AtABI1*, and *AtABF3*), proline biosynthesis (*AtP5CS1*), and ROS scavenging system (*AtSOD (Cu/Zn)*, *AtPOD*, and *AtCAT*) and those associated with drought stress (*AtRD29A*, *AtRD29B*, and *AtDREB2A*) (Fig. 10). These results revealed that the overexpression of *TaWRKY31* led to an increase in the transcript levels of these 12 genes. Our findings suggest that *TaWRKY31* may play a crucial role in the response to drought stress by regulating the expression of stress-related genes.

## Discussion

WRKY TFs, which are one of the largest families of TFs, play a critical role in regulating transcriptional reprogramming associated with plant stress responses. Several WRKY TFs, such as *GhWRKY17* and *GhWRKY21* in cotton [26, 27], *ZmWRKY58* in maize [28], *GmWRKY54* in soybean [29], *IgWRKY50* and *IgWRKY32* in *Iris germanica* [30], *EjWRKY17* in *Eriobotrya japonica* [22], and *ItfWRKY70* in sweet potato [31], have been reported to regulate drought tolerance in various plant species. However, the role of WRKY TFs in mediating the drought response in wheat remains poorly understood. Herein, we cloned the gene encoding a novel WRKY TF belonging to the family group II, *TaWRKY31*, from wheat leaves. The ORF of TaWRKY31 spans a length of 858 bp and encodes 285 amino acid residues. Multiple sequence alignment revealed that TaWRKY31 possesses a highly conserved WRKYGQK heptapeptide at the N-terminus and a C_2_H_2_-type zinc finger motif at its C-terminus. Moreover, phylogenetic analysis demonstrated that *TaWRKY31* shares significant homology with *HvWRKY57* in barley and *AtWRKY57* in *Arabidopsis thaliana*. Previous studies have shown that *AtWRKY57* overexpression enhances drought resistance in rice [32], suggesting a potential association between *TaWRKY31* and drought resistance. In this study, transient transformation of the p35s-1301-*TaWRKY31*-GFP recombinant vector in tobacco leaves revealed a strong GFP signal exclusively in the nucleus, indicating a putative nuclear role for *TaWRKY31*, consistent with the findings of subcellular localization of some other WRKY TFs [33, 34]. Both full-length and N-terminal segments of *TaWRKY31* exhibited normal growth on SD-W/H/A selective medium, suggesting the presence of transcriptional activation activity in the N-terminal region. The expression of the TaWRKY31 TF exhibited variations across different tissues and under different abiotic stress conditions. Our study revealed that *TaWRKY31* gene expression was detected in six wheat organs, namely, flag leaf, glume, lemma, palea, awn, and stem. Notably, the glume exhibited the highest expression level. Previous research has highlighted the crucial role of non-leaf organs in wheat’s drought resistance [35, 36]. Furthermore, *TaWRKY31* displayed distinct expression patterns under different abiotic stress treatments. We noted that the relative expression level of *TaWRKY31* increased at various time points in response to PEG, ABA, NaCl, and SA treatments, while it decreased when the plants were subjected to ethylene treatment. These findings suggest that *TaWRKY31* likely plays a significant role in regulating the response of wheat to various abiotic stresses.

In this study, we successfully generated two *TaWRKY31*-silenced wheat lines, BSMV:*TaWRKY31*-1as and BSMV:*TaWRKY31*-2as, using the BSMV–VIGS technique. The expression levels of *TaWRKY31* in the silenced plants were significantly lower than those in the Mock and BSMV:γ control plants. Additionally, we obtained transgenic *Arabidopsis* lines through the *Agrobacterium*-mediated floral dip method, selecting OE-19 and OE-26 strains as overexpression lines for subsequent experiments based on antibiosis screening and qRT-PCR validation. Under normal conditions, plants maintain a dynamic balance between the production and scavenging of ROS. However, under stress, such as drought, ROS levels increase, thereby resulting in the oxidation of cell membrane lipids, proteins, and nucleic acids [37, 38]. In the present study, following drought treatment, the leaves of *TaWRKY31*-silenced strains exhibited extensive blue staining with NBT, whereas Mock and BSMV:γ control plants showed relatively low levels of O_2_·^–^ accumulation. We also measured H_2_O_2_ content in both wheat and *Arabidopsis* and found that it was significantly higher in the *TaWRKY31*-silenced wheat lines compared to that in Mock and BSMV:γ control plants. Conversely, in the transgenic *Arabidopsis* lines (OE-19 and OE-26), the H_2_O_2_ content exceeded that of the WT and VC plants. Antioxidant enzymes, including SOD, CAT, and POD, play a crucial role in the degradation of excess ROS in plants [39, 40]. We observed decreased SOD, POD, and CAT activities in both BSMV:*TaWRKY31*-1as and BSMV:*TaWRKY31*-2as wheat strains compared to that in Mock and BSMV:γ control plants. In contrast, compared to WT and VC plants, the transgenic *Arabidopsis* lines exhibited higher SOD, POD, and CAT activities. Consistent with the H_2_O_2_ results, we found that the MDA content in both wheat and *Arabidopsis* followed the same trend. As reported by Schroeder et al., when plants are subjected to drought stress, the RWC and water potential of leaves are reduced, which leads to water loss in guard cells, causing plants to partially or fully close their stomata to prevent excessive water loss from leaves [41]. Herein, compared to WT plants, *TaWRKY31*-silenced strains showed a greater decrease in RWC. The stomatal closure rate of *Arabidopsis* overexpressing *TaWRKY31* was higher than that of the WT and VC lines under PEG-6000-simulated drought stress. This finding is consistent with the results of the WLR observed in isolated leaves. Taken together, these physiological indicators suggest that silencing *TaWRKY31* significantly reduced drought stress tolerance in wheat, whereas overexpressing *TaWRKY31* enhanced drought resistance in *Arabidopsis*.

Silencing *TaWRKY31* in wheat and overexpressing it in *Arabidopsis* under drought conditions led to changes in both physiological parameters and gene expression profiles. In the BSMV:*WRKY31*-1as and BSMV:*WRKY31*-2as wheat lines, the expression of ROS scavenging system-related genes (*TaSOD (Fe)*, *TaPOD*, and *TaCAT*) decreased after drought treatment. Conversely, in *TaWRKY31* overexpressing *Arabidopsis*, the expression of *AtSOD (Cu/Zn)*, *AtPOD*, and *AtCAT* increased. DREB TFs play a crucial role in enhancing abiotic stress tolerance in plants by regulating the expression of stress-inducible genes through interaction with DRE/CRT cis-elements [42]. Overexpression of the *DREB2A* gene enhances drought tolerance in *Arabidopsis* [43], while *TaDREB1* and *TaDREB3* are involved in drought tolerance regulation in wheat [44, 45]. In this study, the expression levels of *TaDREB1* and *TaDREB3* decreased, while *AtDREB2A* expression increased. Free proline plays a crucial role in regulating the osmotic potential of plants. Under drought, salt, and cold stress conditions, the expression of Δ1-pyrroline-5-carboxylate synthase (P5CS), a key enzyme in the proline biosynthetic pathway, increases. This leads to the accumulation of significant quantities of proline in plant cells. Yang et al. reported that *SpP5CS* overexpressing *Arabidopsis* is more tolerant to drought stress than the WT [46], whereas the deletion mutant *sp5cs* is less resistant to drought stress. The qRT-PCR analysis revealed a decrease in *TaP5CS* expression level, with an increase in the expression level of *AtP5CS1*. ABA, which plays a significant role in plant adaptation to environmental stresses such as drought, cold or high salt stress [47], is synthesized through a key step involving 9-cis-Epoxy carotenoid dioxygenase (NCED) catalyzed carotenoid cleavage. The resulting product, xanthoxin, is further converted to abscisic aldehyde by a short-chain dehydrogenase/reductase encoded by *ABA2* [48, 49]. Overexpression of *AtNCED3* has been shown to improve drought tolerance in soybean [50]. In our study, the expression of *AtNCED3* and *AtABA2* was upregulated in both OE-19 and OE-26 strains, while *TaNCED1* expression decreased in *TaWRKY31*-silenced wheat. Additionally, we examined the expression patterns of key genes in the ABA signaling pathway. *TaWRKY31* overexpressing plants showed upregulation of *AtSnRK2.2*, *AtABI1*, and *AtABF3*, while gene-silenced wheat exhibited downregulation of *TaSnRK2* and *TaPYL5*. The expression levels of drought-stress associated genes, *AtRD29A* and *AtRD29B*, were significantly higher in the transgenic *Arabidopsis* lines than in the WT and VC strains. Taken together, these findings indicate that *TaWRKY31* regulates plant drought resistance by modulating the expression of related genes.

## Conclusion

The TaWRKY31 TF, isolated and characterized from wheat, is localized in the nucleus, and its N-terminal region (1-126aa) possesses transcriptional activation activity. The expression of *TaWRKY31* is induced by six abiotic stresses, including PEG-6000, NaCl, and ABA. To investigate the function of *TaWRKY31* in stress response, two wheat plants with silenced *TaWRKY31* expression (BSMV:*TaWRKY31*-1as and BSMV:*TaWRKY31*-2as) were generated using the VIGS technique. *TaWRKY31* gene silencing resulted in decreased activities of SOD, POD, and CAT, while increasing the levels of MDA and ROS. Additionally, the expression of stress response genes was suppressed in the silenced parts. Further investigations involved overexpressing *TaWRKY31* in transgenic *Arabidopsis* plants, which led to increased activities of SOD, POD, and CAT, decreased levels of MDA and ROS, and activation of stress response gene expression. Compared to WT plants, the transgenic plants exhibited reduced stomatal opening but had relatively longer roots under drought treatment. Our findings indicate the promising role of *TaWRKY31* as a positive regulator of drought stress in wheat, demonstrating its potential application in molecular breeding programs aimed at enhancing drought resistance in wheat.

## Materials and methods

### Plant materials, growth conditions, and treatments

To initiate the experiment, seeds of *Arabidopsis* ecotype *Columbia* (Wild type, WT) and overexpression lines were subjected to sterilization using a 10% (w/v) sodium hypochlorite (NaClO) solution for 15 min, washed six times with sterilized deionized water, and then vernalized for 3 d at 4℃ under shaded light conditions. Subsequently, sterilized seeds were carefully placed on 1/2 MS medium supplemented with 1% (w/v) agar and adjusted to a pH of 5.8. Furthermore, for the drought tolerance analysis experiment at adult stage, the seedlings were initially grown on 1/2 MS medium for approximately 10 d and then transplanted into pots filled with a substrate consisting of a 1:1 mixture of soil and vermiculite. The plants were cultivated in a growth chamber maintained at 22℃, with a light intensity of 180 µmol·m^− 2^·s^− 1^ and a photoperiod of 16 h/8 h light/dark cycle), for 4 weeks. Prior to conducting the drought treatment, the pots were thoroughly watered, after which the watering was withheld for several days. Throughout the drought treatment period, daily photographs of the plants were taken. For physiological measurements, plant leaves subjected to a natural drought for a duration of 8 d were selected.

To investigate the expression of *TaWRKY31*, a winter wheat variety called Pubing143 (known for its drought insensitivity) was utilized. The wheat seeds were initially subjected to surface sterilization using 75% ethanol (v/v) for 15 min, rinsed six times with distilled water, and subsequently germinated on a wet filter paper at 22℃ for 3 d. Once germinated, the seeds were transferred to pots filled with a 1/2 Hoagland solution under conditions similar to those used for *Arabidopsis*. The 1/2 Hoagland solution was refreshed every 2 d. When the seedlings reached the two-leaf stage at 8 d, they were subjected to six different treatments: drought, high salt, ABA, salicylic acid (SA), low temperature, and ethylene. The treatments were carried out using solutions containing 20% polyethylene glycol (PEG) for drought simulation, 200 mmol·L^− 1^ NaCl for high salt conditions, 100 µmol·L^− 1^ ABA, 500 µmol·L^− 1^ SA, 100 µL·L^− 1^ ethylene, and 4℃ for low-temperature treatment. Leaves were collected from at least three seedlings at specific time intervals (0, 1, 3, 6, 12, 24, and 48 h) after the initiation of each treatment. To perform organ-specific expression analysis, potted wheat plants were grown, and tissue samples were collected from various plant parts including flag leaves, palea, lemma, glumes, awn, and stem from seedlings. The tissue sampling was performed using five different plants, and each sample was collected in triplicate. Immediately after collection, the tissue samples were frozen in liquid nitrogen at − 80℃ to preserve the RNA prior to extraction.

### Isolation and bioinformatics analysis of the *TaWRKY31* gene

To obtain the open reading frame (ORF) sequence of *TaWRKY31*, we retrieved the sequences from Plant TFDB (planttfdb.gao-lab.org) and Ensembl Plants (plants.ensembl.org). We used the NCBI database (www.ncbi.nlm.nih.gov) sequence matching tool (blastn) to search for homologous genes in other species and download the sequences with high similarity. Multiple sequence alignments were generated using DNAMAN software (Lynnon Biosoft, San Ramon, CA, USA). To construct phylogenetic tree, we utilized the MEGA-X software and applied the neighborhood-joining method with a bootstrap value of 1000. The exon–intron structure of *TaWRKY31* was obtained from Gene Structure Display Server 2.0 [20].

### Total RNA extractions, reverse transcription PCR, and quantitative real-time PCR analysis

Total RNA samples from *Arabidopsis* and wheat organs and wheat seedlings, to study the expression of *TaWRKY31* under different abiotic stress conditions, were prepared using TRIzol reagent. The quality of total RNA was assessed using a NanoDrop-1000 spectrophotometer (Thermo Fisher Scientific, Waltham, MA, USA). To synthesize first-strand cDNA, reverse transcription PCR was performed on 1 µg of total RNA using Evo M-MLV RT Mix Kit with gDNA Clean for qPCR (Accurate Biotechnology, Changsha, China). Quantitative Real-Time PCR (qRT-PCR) was conducted using the SYBR® Green Premix Pro Taq HS qPCR Kit with the CFX96 real-time system to detect the expression levels of related genes in the samples. The *AtTubulin* gene was used as the internal reference gene in *Arabidopsis thaliana*, while the *TaEF-1a* gene served as a reference gene in wheat [51]. The specific primers utilized are listed in Supplementary Table 1. The qRT-PCR analysis was based on data from three samples, each containing three technical replicates. The 2^−∆∆CT^ method was used to estimate the relative expression levels and ensure the accuracy of the results.

### Plasmid construction and transformation of *TaWRKY31* in *Arabidopsis*

The full coding sequence of *TaWRKY31* was amplified using specific primers (Supplementary Table S1). The purified PCR product was then inserted into the pBI111L vector. The recombinant vector was introduced into the *Agrobacterium* strain GV3101, and the *TaWRKY31* gene was transformed into *Arabidopsis* using the floral dip method [52]. Seeds from infiltrated plants were harvested and cultivated on 1/2 MS medium supplemented with 50 µg·mL^− 1^ kanamycin for selection. Kanamycin-resistant plants were transferred to soil one week after germination and grown under controlled conditions [19]. T3 transgenic progeny were generated after two generations of selfing, and the transcriptional levels of *TaWRKY31* in the overexpression (OE) lines were verified by PCR and qRT-PCR [20].

### Subcellular localization analysis of *TaWRKY31*

For the subcellular localization analysis of *TaWRKY31*, we designed primers with appropriate double restriction enzyme sites, *Xba*I and *Kpn*I, based on the full-length *TaWRKY31* and p35s-1301-GFP vector. The target gene sequence was amplified using these primers. The complete *TaWRKY31* ORF without the termination codon was then inserted into the p35s-1301-GFP vector using the ClonExpressII One Step Cloning Kits (Vazyme, Nanjing, China). The p35s-1301-*TaWRKY31*-GFP plasmid and p35s-1301-GFP empty vector were transformed into *Agrobacterium* GV3101 and subsequently infiltrated into the abaxial surface of *Nicotiana benthamiana* leaves using 1-mL needleless syringes along with NLS-mCherry and P19 as controls [53]. The transformed tobacco plants were initially grown in a dark environment at 22℃ for 24 h and then transferred to controlled conditions with a temperature of 22℃, a photoperiod of 16 h/8 h, and an illumination intensity of 180 µmol·m^− 2^·s^− 1^ for 2 d. Green fluorescent protein (GFP) signals were observed using a laser confocal microscope (Andor, Belfast, UK) at an excitation wavelength of 488 nm. The specific primers used for this analysis are listed in Supplementary Table S1.

### Analysis of transcriptional activation in yeast

The TaWRKY31 protein is composed of 285 amino acid residues, with TaWRKY31 TF belonging to II group of WRKY TFs. The region spanning amino acid residues 127 to 185 contains the complete WRKY domain and the C_2_H_2_ zinc finger motif. Specific primers, listed in Supplementary Table S1, were designed to amplify different fragments of TaWRKY31:TaWRKY31-N(1-126aa), TaWRKY31-NW(1-185aa), TaWRKY31-WC(127-285aa), TaWRKY31-C(186-285aa), and the full-length TaWRKY31(1-285aa). The complete TaWRKY31 ORF and the four truncated fragments were inserted into the *Eco*RI and *Bam*HI sites of pGBKT7 vector. The empty pGBKT7 vector was used as a control. The recombinant vectors were then introduced into the yeast strain AH109 using the PEG-LiAC method. The transformed yeast cells were grown on selective medium (SD) without tryptophan (SD-W) and screened by PCR. Positive clones were identified on SD-W medium and further confirmed on SD-W medium lacking tryptophan, histidine, and adenine (SD-W/H/A). Additionally, the transcriptional activation activity of the TaWRKY31 protein was assessed by performing an X-α-D-galactosidase (X-α-gal) assay on SD-W/H/A medium at 28℃. The transcriptional activation activity was observed after 3 d of incubation [20].

### Virus-induced gene silencing mediated by BSMV

To investigate the potential role of *TaWRKY31* in the abiotic stress response in wheat, we employed the barley stripe mosaic virus (BSMV)–virus-induced gene silencing (VIGS) system to knock down the expression of *TaWRKY31*. Two fragments of different lengths within the *TaWRKY31* ORF, namely *TaWRKY31*-1as and *TaWRKY31*-2as, were selected for this purpose. The γ-*TaWRKY31*-1as and γ-*TaWRKY31*-2as vectors were generated by subcloning *TaWRKY31*-1as and *TaWRKY31*-2as fragments into the γ vector using the *Pac*I and *Not*I restriction sites. The ClonExpressII One Step Cloning Kits (Vazyme, Nanjing, China) were utilized for this cloning procedure. The vectors, including α, β, γ, γ-TaPDS, γ-*TaWRKY31*-1as, and γ-*TaWRKY31*-2as, were linearized using different enzymes and transcribed into RNA using the RiboMAX™ Large Scale RNA Production System and Ribo m^7^G Cap Analog Kits (Promega, Madison, WI, USA). To initiate the virus-induced gene silencing, RNAα and RNAβ were mixed in a 1:1:1 ratio with transcripts of γ, γ-TaPDS, γ-*TaWRKY31*-1as, and γ-*TaWRKY31*-2as in FES buffer consisting of 0.1 M glycine (pH 8.9), 0.06 M K_2_HPO_4_, 1% w/v bentonite, 1% w/v sodium pyrophosphate, and 1% w/v celite [51, 54]. The resulting BSMV viruses, namely BSMV:γ, BSMV:*TaPDS*, BSMV:*TaWRKY31*-1as, and BSMV:*TaWRKY31*-2as, were inoculated onto the second fully expanded leaves of wheat seedlings. This inoculation was performed by gently rubbing the leaf surface with a gloved finger and maintained at 25℃ [55–57]. As a positive control, BSMV:TaPDS (targeting the wheat phytoene desaturase) was included, while 1 × FES buffer (Mock) served as a negative control [51, 58]. After observing and capturing the virus phenotype (photobleaching of the positive control) on day 10 post-BSMV treatment, the wheat seedlings were subjected to drought treatment.

### Seed germination rate and root length assay

In order to investigate the effects of *TaWRKY31* on seed germination, *Arabidopsis* seeds from homozygous T_3_ transgenic lines of *TaWRKY31* and WT seeds were sterilized in 10% (v/v) sodium hypochlorite (NaClO) for 15 min, rinsed six times with sterilized distilled water, and then kept at 4℃ in the dark for 3 d. Afterward, the seeds were sown on 1/2 MS solid medium supplemented with 0, 150, and 300 mM mannitol, along with 1.0% (w/v) agar. The germination rate of seeds was recorded daily for a period of 5 d. Each replicate consisted of at least 50 seeds, and three parallel replicates were performed. This germination rate measurement experiment was repeated three times.

For the determination of *Arabidopsis* root length under different treatment experiments, *Arabidopsis* seeds (both WT and transgenic strains) were uniformly planted on 1/2 MS solid medium containing different concentrations of mannitol. The root length of the seedlings was measured 8 d post-germination. Three biological replicates were performed for each treatment, with 10 seeds (plants) per replicate.

### Water loss rate and relative water content

To evaluate WLR and relative water content (RWC), WT, vector control (VC), and *TaWRKY31*-overexpressing T_3_*Arabidopsis* seeds were cultivated in a growth chamber (22℃, 180 µmol·m^− 2^·s ^− 1^, and 16 h/8 h day/night photoperiod) and watered thoroughly 1 d before the experiment. Leaves from more than five plants in the same state were excised, weighed immediately, and placed on filter paper at room temperature (~ 25℃) for 0, 1, 2, 3, 4, 5, 6, and 12 h. The leaves were weighed at the designated time points, and the WLR was subsequently calculated. Three replicates were performed for each line [59].

Furthermore, the fourth leaf of silenced wheat line was collected, and its fresh weight (FW) was immediately measured. The wheat leaves were then soaked in distilled water (protected from light) for 4 h and weighed (total weight, TW), after which they were dried in an oven at 80℃ until a constant weight was achieved and weighed again (dry weight, DW).

### Stomatal aperture assays

To assess the stomatal aperture, the lower epidermis layers of leaves from 3-week-old *Arabidopsis* seedlings (WT, VC, and transgenic lines) were collected. The leaves were floated on a stomatal opening solution (10 mM KCl, 0.2 mM CaCl_2_, and 10 mM MES-KOH; pH, 6.15) for 2.5 h to induce stomatal opening. Then, the lower epidermis of leaves was placed in an opening solution with 7.5% PEG6000 and treated in a growth incubator for 2.5 h [60]. The stomata were observed and photographed using a microscope, and the width and length of 50 stomata per line were measured. The stomatal aperture, defined as the ratio of width to length, was calculated. All experiments were repeated three times.

### Measurements of physiological index related to stress tolerance

To assess the accumulation of superoxide anion radicals (O_2_·^−^) in seedling leaves after stress treatment, histochemical staining with nitro blue tetrazolium (NBT) was performed, as described by Fryer et al. [61]. The determination of hydrogen peroxide content in the leaves was carried out using a hydrogen peroxide assay kit (Nanjing Jiancheng). The content of MDA in the leaves was determined using the thiobarbituric acid method. The quantification of soluble protein in plant tissues was performed using the Coomassie brilliant blue solution. The activity of SOD was detected by monitoring the inhibition of photochemical reduction of NBT. The activity of peroxidase activity (POD) was estimated using the method described by Lv et al. [62]. The activity of CAT was determined according to the method described by Aebi [63].

### Statistical analysis

The data were first analyzed using Microsoft Office Excel 2013. The error bars in the figures represent the standard error (SE). The significance level was determined using the Student’s t-test method, with * indicating *p* < 0.05, ** indicating *p* < 0.01, *** indicating *p* < 0.001, and **** indicating *p* < 0.0001. Statistical analysis was performed using SPSS Statistics 20.0 software. The figures were generated using GraphPad Prism 7 software.

### Electronic supplementary material

Below is the link to the electronic supplementary material.

Supplementary Material 1

## Data Availability

The datasets generated during the current study are available in the Plant TFDB repository (planttfdb.gao-lab.org; transcript ID: Traes_2BL_A69F6C5DF.1).

## Abbreviations

Ta: *Triticum aestivum* L
VIGS: Virus-induced gene silencing
TFs: Transcription factors
MDA: Malondialdehyde
SOD: Superoxide dismutase
POD: Peroxidase
CAT: Catalase
REB1: Dehydration responsive element binding 1
P5CS: 1-Pyrroline-5-carboxylate synthetase
NCED1: 9-cis-epoxycarotenoid dioxygenase 1
SnRK2: Sucrose nonfermenting-1-related protein kinase 2
PP2C: Protein phosphatase 2 C
PYL5: ABA receptors 5
ABA2: Xanthoxin dehydrogenase protein 2
ABI1: ABA Insensitive 1
ABF3: ABA-induced protein-ABRE Binding Factor 3
RD29A: Desiccation 29 A
RD29B: desiccation 29B
DREB2A: One of the transcription factors
ABA: Abscisic acid
ORF: Open reading frame
GFP: Green fluorescent protein
PEG: Polyethylene glycol
ROS: Reactive oxygen species
