# Supplementary material for: TaWRKY31, a novel WRKY transcription factor in wheat, participates in regulation of plant drought stress tolerance
Source: BMC Plant Biol. 2024 Jan 3;24:27. doi: 10.1186/s12870-023-04709-7 (PMC10763432; doi:10.1186/s12870-023-04709-7)
Supplement: Supplementary file 1 — Supplementary Material 1 [file 12870_2023_4709_MOESM1_ESM.docx]

Supplementary Material

Enhanced drought tolerance in transgenic Arabidopsis thaliana through overexpression of a novel WRKY transcription factor gene, *TaWRKY31*

Miaomiao Ge^1†^, Yan Tang^1†^, Yijun Guan^1^, Meicheng Lv^1^,Chunju Zhou^1^, Huiling Ma^1*^and Jinyin Lv^1*^

*** Correspondence:** Corresponding Author: hl65@nwafu.edu.cn; [jinyinlv@nwsuaf.edu.cn](mailto:jinyinlv@nwsuaf.edu.cn)

# Supplementary Table

Table S1 Primer information in this study

| Pursose | Gene | Primers (5' - 3') |
| --- | --- | --- |
| qRT-PCR | TaEF-F | TGGTGTCATCAAGCCTGGTATGGT |
|  | TaEF-R | ACTCATGGTGCATCTCAACGGACT |
|  | TaWRKY31-F | CCCTTTCCCAAGGAGCTACTAC |
|  | TaWRKY31-R | AGTCGTTGTACATGAGGTGGTG |
|  | TaSOD (Fe)-F | GGTTGGGTTTGGCTTGTC |
|  | TaSOD (Fe)-R | TCGCCTGTCATCCTTGTAAT |
|  | TaPOD-F | TTGTGGTGGCGGTGGTAGTGG |
|  | TaPOD-R | CGAAGCAGTCGTGGAAGTGGAG |
|  | TaCAT-F | GTGCTGAACCGCAACATCG |
|  | TaCAT-R | AGCAGCTTGTCGTCCGAGT |
|  | TaDREB1-F | CTCCATTGCCGATGTCTA |
|  | TaDREB1-R | GGATACTTCCAGGGTCTTG |
|  | TaDREB3-F | GATTCCGTGGTGTAAGGC |
|  | TaDREB3-R | GTGGTTGGTGGATGTTGTAG |
|  | TaERF3-F | AGCAATCAGGCAAAGCAACC |
|  | TaERF3-R | ACGACTCAGAAGGAACCACGAC |
|  | TaERF4b-F | GACGGCGACGAGAATAGCA |
|  | TaERF4b-R | GAAAGAAGAAACGCAGAGCAGA |
|  | TaP5CS-F | GACAAGTCCCGTGTTGGTAGAG |
|  | TaP5CS-R | CGTGCAGCAACAGCCATTT |
|  | TaNCED1-F | TCATCGCCGTCCACCAGGAA |
|  | TaNCED1-R | TTGAGGCTCTGCCCGTCCTT |
|  | TaSnRK2-F | TGTTTGGTCATGCGGAGTAA |
|  | TaSnRK2-R | GTGATCGTTTTGCGGAAGTT |
|  | TaPP2C-F | AGGGTGGCAAGGTCATACAG |
|  | TaPP2C-R | ATTGTGACCTCTGGGACAGG |
|  | TaPYL5-F | GTGGTGGAGTCGTACGTGGT |
|  | TaPYL5-R | GTTGCACTTGACGATGGTGT |
|  | AtTubulin-F | AAGGGACACTACACGGAAGGA |
|  | AtTubulin-R | GGAACACCGAGAAGGTAAGCA |
|  | AtRD29A-F | CTTGATGGTCAACGGAAGGT |
|  | AtRD29A-R | CAATCTCCGGTACTCCTCCA |
|  | AtRD29B-F | AGAAGGAATGGTGGGGAAAG |
|  | AtRD29B-R | CAACTCACTTCCACCGGAAT |
|  | AtDREB2A-F | TGACCTAAATGGCGACGATGT |
|  | AtDREB2A-R | TCCAAGTAACTCAAGTCGTCG |
|  | AtPOD1-F | TCTCATTACGGAGCACAA |
|  | AtPOD1-R | AAGCCAGTATCTATAAGCAC |
|  | AtSOD (Cu/Zn)-F | TGCCACCTTCACAATCAC |
|  | AtSOD (Cu/Zn)-R | GCTTTAGCCCTGGAGACC |
|  | AtCAT1-F | TGGAGGAGCCAATCACAG |
|  | AtCAT1-R | CAAGACCAAGCGACCAAC |
|  | AtABI1-F | GTTTGGGATGTAATGACGGATG |
|  | AtABI1-R | ACCACACTTATGTTGTCTTTGC |
|  | AtABA2-F | GGGAGGCGTTGGTCCACATTCT |
|  | AtABA2-R | ATCAACCGTCAGTTCCACCCCT |
|  | AtP5CS-F | CTTGTGATACGGATATGGCAAAGCG |
|  | AtP5CS-R | CCTTGGTCCACCATACAAAGTGACTCC |
|  | AtABF3-F | AACGCTGGGAGAGATGACTTTGGA |
|  | AtABF3-R | TCCCAAGACCTCCATTACTGCCAA |
| Subcellular localization | p35S-1301-TaWRKY31-F | gagaacacgggggactctagaATGGCCGGCGTCGAGTGC |
|  | p35S-1301-TaWRKY31-R | gcccttgctcaccatggtaccTCCGTGCCTCATCGCTGG |
| Transcriptional Activation | TaWRKY31-N-F | atggccatggaggccgaattcATGGCCGGCGTCGAGTGC |
|  | TaWRKY31-N-R | ccgctgcaggtcgacggatccGAGGTGGTCAATCTCCGTCTTG |
|  | TaWRKY31-W-F | atggccatggaggccgaattcGAGGACGGATACCGCTGGA |
|  | TaWRKY31-W-R | ccgctgcaggtcgacggatccGGTGTGGTGGCAGTGCTGGCC |
|  | TaWRKY31-C-F | atggccatggaggccgaattcGTCACCTTCCCCCGCGGCGCC |
|  | TaWRKY31-C-R | ccgctgcaggtcgacggatccCTATCCGTGCCTCATCGCTGGAG |
| TaWRKY31 | pBI111L-TaWRKY31-F | CGTCTAGAATGGCCGGCGTCGAGTGCGGCG |
|  | pBI111L-TaWRKY31-R | CGTCTAGACTATCCGTGCCTCATCGCTGGA |
| BSMV-VIGS | TaWRKY31-1as-F | tttttagctagctgattaattaaATGGCCGGCGTCGAGTGC |
|  | TaWRKY31-1as-R | tccgttgctagctgagcggccgcGCGGATCCAGCGGCGGAA |
|  | TaWRKY31-2as-F | tttttagctagctgattaattaaGACCACCTCGAGGACGGATAC |
|  | TaWRKY31-2as-R | tccgttgctagctgagcggccgcGGTGGTGGTGGTGTGCCG |
